# Outcomes of Intrauterine Device Insertion by Certified Midwives and Obstetric Nurse Practitioners

**DOI:** 10.1590/0034-7167-2022-0286

**Published:** 2023-11-27

**Authors:** Thalita Vital Botelho, Ana Luiza Vilela Borges

**Affiliations:** I Universidade de São Paulo. São Paulo, São Paulo. Brazil

**Keywords:** Intrauterine Device, Nurse, Midwife, Contraception, Family Planning, Dispositivo Intrauterino, Enfermeras Ostetrices, Matrona, Anticoncepción, Planificación Familiar, Dispositivo Intrauterino, Enfermeira Obstetra, Obstetriz, Contracepção, Planejamento Familiar.

## Abstract

**Objectives:**

to evaluate the outcomes of Interval Copper Intrauterine Device (IUD) insertion performed by certified midwives and obstetric nurse practitioners at a Peri-Hospital Birth Center.

**Methods:**

a cross-sectional study was conducted involving 75 women who underwent IUD insertion between January 2018 and February 2020. Data collection was carried out using medical records and telephone interviews.

**Results:**

no instances of uterine perforation were observed. Expulsion rates of the devices were 1.3% within 30 to 45 days of use and 5.3% within the first year of use. The follow-up removal rate was 4.0%. The average pain score reported was 4.2 (SD = 3.3). Among those who continued using the device, 93.1% expressed satisfaction.

**Conclusions:**

the findings demonstrate that IUD insertion by certified midwives and obstetric nurse practitioners is a safe procedure, yielding outcomes comparable to those reported in the existing literature.

## RESUMO

**Objetivos:**

avaliar os desfechos da inserção do Dispositivo Intrauterino de Cobre de Intervalo por obstetrizes e enfermeiras obstetras em um Centro de Parto Normal Peri-hospitalar.

**Métodos:**

estudo transversal conduzido com 75 mulheres que tiveram o dispositivo inserido entre janeiro de 2018 e fevereiro de 2020. A coleta de dados foi realizada a partir de prontuários e contato telefônico.

**Resultados:**

não houve nenhuma perfuração uterina; 1,3% dos dispositivos foram expulsos com 30 a 45 dias de uso e 5,3% no primeiro ano de uso; a taxa de necessidade de remoção no retorno foi de 4,0%; e a pontuação média de dor foi 4,2 (DP = 3,3). Entre aquelas que ainda usavam o dispositivo, 93,1% se consideraram satisfeitas.

**Conclusões:**

a inserção do dispositivo por enfermeiras obstetras e obstetrizes mostrou ser segura, com desfechos similares aos observados na literatura.

## RESUMEN

**Objetivos:**

evaluar los resultados de la inserción del dispositivo intrauterino de cobre (DIU) realizado por matronas certificadas y enfermeras obstétricas especializadas en un Centro de Nacimientos Peri-Hospitalario.

**Métodos:**

se realizó un estudio transversal que incluyó a 75 mujeres sometidas a la inserción del DIU entre enero de 2018 y febrero de 2020. La recopilación de datos se realizó mediante registros médicos y entrevistas telefónicas.

**Resultados:**

no se observaron casos de perforación uterina. Las tasas de expulsión del dispositivo fueron del 1,3% en los primeros 30 a 45 días de uso y del 5,3% durante el primer año de uso. La tasa de retirada durante el seguimiento fue del 4,0%. La puntuación promedio del dolor informada fue de 4,2 (DE = 3,3). Entre aquellas que continuaron utilizando el dispositivo, el 93,1% manifestó satisfacción.

**Conclusiones:**

los hallazgos demuestran que la inserción del DIU por parte de matronas certificadas y enfermeras obstétricas especializadas es un procedimiento seguro, que produce resultados comparables a los reportados en la literatura existente.

## INTRODUCTION

Cleland et al.^(1)^ have calculated that over the past 20 years, the increased utilization of modern contraceptive methods has resulted in a reduction of maternal mortality rates by approximately 40% in lowand middle-income countries. It is evident that access to modern contraceptive methods has contributed to the prevention of unintended pregnancies and unsafe abortions, both of which are significant contributors to maternal mortality, particularly in countries with restrictive abortion laws, such as Brazil. In 2014, Singh et al.^(2)^ estimated that if all women desiring to avoid pregnancy were to employ a modern contraceptive method, unintended pregnancies could be reduced by 70%.

The National Health Survey reveals that contraceptive usage in Brazil continues to revolve around three primary methods: oral contraceptive pills, male condoms, and female sterilization^(3)^. The first two methods are categorized as short-acting^(4)^, relying on user compliance for efficacy and contraceptive impact^(5)^. Long-acting reversible contraceptives (LARCs) are currently underutilized. Despite being the second most commonly employed reversible contraceptive globally, with a prevalence of 17%^(6)^, the intrauterine device (IUD) was utilized by only 1.9% of Brazilian women in 2013^(7)^ and 4.4% in 2019^(3)^. Regional studies corroborate these findings, reporting IUD usage rates of approximately 3% in Rio Grande do Sul in 2015^(8)^ and 2.5% in São Paulo during the same year^(9)^.

Although LARCs are underutilized in Brazil, they offer higher contraceptive efficacy compared to short-acting methods, with an annual pregnancy rate of less than 1%^(10)^. This justifies the widespread availability of LARCs, including the copper intrauterine device (Tcu-380A), as recommended by reputable organizations such as the Ministry of Health, the Center for Disease Control and Prevention, the American Academy of Pediatrics, the World Health Organization (WHO), and the Brazilian Federation of Gynecology and Obstetrics Associations^(5,10-15)^. Notably, the copper IUD remains the sole LARC widely accessible in primary healthcare within the Unified Health System.

The IUD is regarded as an excellent choice for women seeking long-acting reversible contraception, including postpartum women. It can be safely utilized by nulliparous women, adolescents, and during the perimenopausal period without adverse health effects. Potential complications associated with the use of the copper IUD include uterine perforation, an infrequent occurrence with rates ranging from 0.13% to 0.22%, and expulsion, with rates of up to 10% within the first year of use^(16)^.

In addition to the limited availability of the method, research reports various barriers that contribute to its underutilization, such as the lack of necessary supplies for insertion, healthcare professionals’ limited knowledge regarding eligibility criteria, absence of care protocols in healthcare services, long wait times and appointment scheduling requirements, mandatory participation in prerequisite groups, and unnecessary examination requests^(17-19)^. The World Health Organization (WHO) also indicates that the number of trained healthcare professionals for insertion is insufficient and recommends involving other professional categories in counseling and provision of contraceptive methods, including the IUD^(20)^.

In Brazil, certified midwives and nurses have been legally recognized as qualified professionals to prescribe and insert the copper IUD since 2010, as stated in Opinion No. 17/2010 from the Federal Nursing Council^(21)^. In 2018, the Ministry of Health published a technical manual for healthcare professionals on the copper IUD, highlighting the importance of nursing involvement in this care^(15)^. However, in December 2019, the Ministry of Health issued Technical Note 38/2019-DAPES/SAPS/MS^(22)^, revoking the previous Technical Note No. 5/2018-CGSMU/DAPES/SAS/MS, which recognized nurses and midwives as capable of IUD insertion^(15)^. This study is conducted within the context of an intense debate in the country regarding the legal competence of nurses and midwives to insert and manage the IUD. Accordingly, the study aims to evaluate the outcomes of interval copper IUD insertion by midwives and obstetric nurses at a Peri-Hospital Birth Center (PHBC).

## OBJECTIVES

To evaluate the outcomes of Interval Copper Intrauterine Device (IUD) insertion by midwives and obstetric nurses at a Peri-Hospital Birth Center.

## METHODS

### Ethical aspects

The research was conducted in accordance with the guidelines of Resolution 466/12^(23)^ and received approval from the Research Ethics Committee of the School of Public Health at the University of São Paulo. Participants were contacted via WhatsApp to introduce the research and its objectives, and a digital copy of the Informed Consent Form (ICF) was provided for them to review and address any questions. Subsequently, authorization was requested to establish contact with the researcher, and a telephone appointment was scheduled. All women provided their consent to participate in the study through the ICF.

### Study design, period, and location

This was a quantitative cross-sectional study conducted following the STROBE guidelines. It took place at a PHBC situated in the southern region of São Paulo city. IUD insertions were performed by obstetric nurses and midwives in women who selected this method, starting from four weeks postpartum, or even those who did not receive prenatal care at the facility and were not in the late postpartum period. Hence, this study pertains to Interval IUD, which is inserted on an outpatient basis. Follow-up monitoring appointments were scheduled for 30 to 45 days after insertion.

### Population or Sample; inclusion and exclusion criteria

The study population consisted of all women who underwent IUD insertion by professionals at the PHBC during the study period. The inclusion criteria included being 18 years of age or older at the time of the telephone interview, having attended the follow-up appointment at the facility, and having had the copper IUD inserted at least 6 months prior to the interview due to the higher incidence of side effects during that period^(10)^. Women who were current or former employees of the institution were excluded from the study to minimize potential bias and the risk of data overestimation due to their familiarity with the services provided.

### Study Protocol

The selection of women included in this study was based on a database from the institution. Therefore, sociodemographic information such as age and education, reproductive history, contraceptive history, as well as data on IUD insertion and follow-up appointments were collected from medical records of eligible women who had their copper IUD inserted between January 2018 and February 2020.

At the scheduled date and time, the researcher contacted the participants, obtained verbal consent, and conducted the interview using a structured questionnaire with an approximate duration of 15 minutes. The questionnaire focused on collecting data regarding expulsion, removal, satisfaction, discontinuation of use, and also included the administration of the Numeric Verbal Pain Scale (NVPS)^(24)^ to assess the level of pain during IUD insertion. The researcher developed the instrument for data collection and management using Google Forms.

### Data analysis and statistics

The data were analyzed using Stata 15.0 software, and reinsertions of the IUD were excluded from the analysis as they were considered secondary outcomes of the intervention. Descriptive analysis was utilized to examine the findings, and the results were reported using absolute numbers, proportions, means, and standard deviations, which are presented in tables and figures. The evaluated outcomes encompassed the perforation rate, IUD expulsion rate, removal rate, and reasons for removal, discontinuation rate and primary reasons, satisfaction with the method among users who continued its use, and the level of pain assessed using the NVPS, where scores of 0-2 represent mild pain, 3-7 indicate moderate pain, and 8-10 correspond to severe pain.

## RESULTS

A total of 75 women participated in the study. The mean age at the time of IUD insertion was 28.2 years (SD = 5.8), with a minimum age of 18 years and a maximum age of 41 years. The majority self-identified as Black (54.7%), were in a relationship (86.7%), had completed higher education (44.0%) or high school (44.0%), were employed (64.0%), and received prenatal care at the institution (73.3%). The sociodemographic characteristics of the women can be observed in Table 1.

**Table 1:** Characterization of women according to age, skin color, marital status, education, and employment status, São Paulo, São Paulo, Brazil, 2020

Variable	n	%
Age (years)		
<25	19	25.3
25-29	31	41.3
≥30	25	33.4
Skin color		
White	34	45.3
Black	41	54.7
Marital status		
Single	10	13.3
In a relationship	65	86.7
Education		
Elementary school or less	9	12.0
Completed high school	33	44.0
Completed higher education	33	44.0
Employment status		
Total	75	100.0

Regarding reproductive history, half of the women had a prior pregnancy (54.7%), the majority had a vaginal delivery (74.7%), and the majority did not report a history of abortion (89.3%); 94.7% also did not report a history of pelvic uterine surgery. As for the menstrual cycle prior to IUD insertion, 69.0% of the women considered their menstrual flow to be moderate. One-third considered their menstrual cramps to be mild (33.3%).

More than half of the participants indicated that they shared the decision to insert the IUD with someone (62.7%). When asked how they became aware of the method, one-third stated that they learned about it through the institution itself (31.2%), during the prenatal and postpartum care period. Media sources also played a significant role (22.1%).

Regarding the contraceptive method used prior to IUD insertion, 49.3% of women reported using male condoms, 22.2% used oral hormonal contraceptives, and 13.3% practiced withdrawal. Only two women had previously used the copper IUD, with expulsion being the main reason for discontinuation.

The majority of users reported receiving contraceptive counseling (98.7%) during insertion. Among those who received this counseling, nearly all considered it sufficient to feel confident with the IUD (98.6%). Slightly over half of the women were accompanied during the IUD insertion (56.0%), with the partner being the most common companion (56.0%).

**Figure 1:**
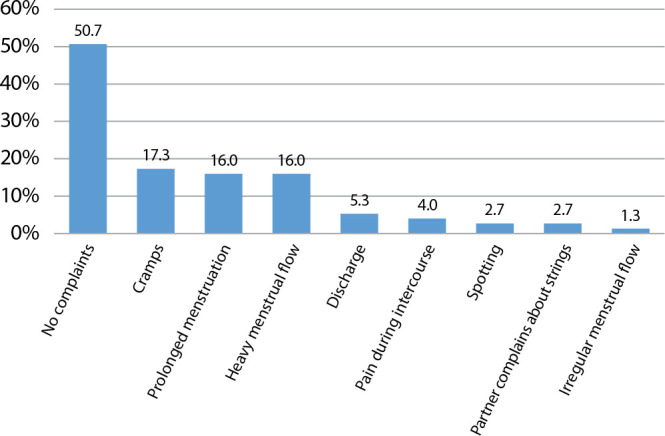
Complaints Reported during the Follow-up Appointment regarding Adaptation to the IUD, São Paulo, São Paulo, Brazil, 2020

When the NVPS was used to measure the level of pain experienced during the insertion procedure, the average score was 4.2 (SD = 3.3), ranging from zero to ten. 37.3% rated the pain as mild (NVPS 0-2), 44.0% as moderate (NVPS 3-7), and 18.7% as severe (NVPS 8-10). During the follow-up appointment, 49.3% of women reported complaints related to adapting to the IUD (Figure 1). Among the reported complaints, the majority were related to the menstrual cycle, such as cramps (17.3%), prolonged menstruation (16.0%), and heavy menstrual flow (16.0%). It is worth noting that only 11 women had more than one complaint. The use of oral anti-inflammatory medication for dysmenorrhea control was necessary in 70.8% of cases.

Three women underwent IUD removal during the follow-up appointment, one due to personal request, one due to misplacement, and the last one due to expulsion (the IUD was found in the cervix). Therefore, the expulsion rate within 30 to 45 days of use was 1.3%. No cases of uterine perforation were reported among the study population.

Routine ultrasonography examinations were not performed, but there were indications for its use in 14 cases. The reasons included patient’s request (n=8), non-visualization of threads (n=2), intense pelvic pain (n=2), suspected misplacement (n=1), and increased bleeding (n=1). Among the ultrasound results, only eight users had the results documented in their medical records. In six cases, the IUD was confirmed to be correctly positioned, one case showed low insertion of the IUD, and one case showed misplacement.

During the telephone interview, it was observed that 77.3% were still using the IUD. Among the women who discontinued its use by the time of the interview, the main reasons were changes in menstrual flow (35.3%) and expulsion (23.5%).

All of the interviewed women, including those who discontinued the use, reported that they would recommend the IUD to friends and family, and 87.0% expressed willingness to use the method again. In terms of satisfaction with the use of the IUD, considering only the women who continued to use the method at the time of the interview (n=58), 93.1% reported being satisfied, and 84.5% believed that their partners were also satisfied. The main results are presented in Table 2.

**Table 2:** Outcomes of IUD insertion by obstetric nurses and midwives, São Paulo, São Paulo, Brazil, 2020

Variable	n	% or mean (SD)
Uterine perforation	0	-
Expulsion within 30 to 45 days of use	1	1.3
Expulsion within the first year of use	4	5.3
IUD removal at follow-up	3	4.0
Level of pain	-	4.2 (3.3)
Satisfaction with IUD use	54	93.1
Total	75	100.0

## DISCUSSION

The results demonstrate that in a healthcare setting where all IUD insertions were performed by nurses and obstetricians, there were no differences in outcomes compared to other studies evaluating insertions conducted by physicians. These findings contribute to the existing body of research conducted in Brazil, Tanzania, India, Australia, Sri Lanka, Kenya, Nepal, and Bangladesh^(25-30)^.

Legal competence for IUD insertion varies significantly across countries. For instance, in Tanzania, interval IUD insertions by obstetricians are already a well-established procedure in healthcare services. Since December 2015, the International Federation of Gynecology and Obstetrics has implemented a training program for postpartum IUD insertion in the country, empowering obstetricians to provide this service^(28)^. In Australia, a study involving 91% of insertions performed by nurses reported a 2% expulsion rate and a 4% device removal rate, with no cases of uterine perforation^(25)^. These data corroborate the consistency of findings in this study, even when considering diverse countries with different realities and healthcare systems.

While the observed expulsion rate in the first year of use in this study was higher than in other studies conducted in various contexts, it falls within the expected range of up to 10%^(16,31-33)^. Thus, the observed percentage in this study can be considered acceptable.

Pain is an underexplored outcome in research on intrauterine devices. However, most studies examining this variable evaluate insertions performed by physicians, which prevents conclusive statements on whether procedures conducted by nurses or obstetricians result in higher or lower pain sensations for users. Nevertheless, the majority of women in this study rated the pain of interval copper IUD insertion as moderate. A study conducted in Portugal assessed factors related to anxiety and pain during hormonal IUD insertion performed by physicians. The NVPS administered immediately after insertion yielded a mean score of 4.7 (SD=2.6)^(34)^. Although similar to the findings in this study, the classification of pain as intense and moderate was higher. Nonetheless, caution should be exercised when comparing these studies due to differences in the analyzed devices and the timing of pain assessment during the post-insertion period.

In this study, the analyzed insertions were performed at various points in the menstrual cycle, and the use of non-steroidal anti-inflammatory drugs before the procedure was left to the discretion of the women. Approximately half of the users were accompanied, which may have contributed to a reduced perception of pain. Another significant aspect is related to the contraceptive counseling received. It is possible that prior information about the procedure provided greater security and comfort to the users, thus impacting their pain experience. The environment may also be relevant in this regard, as the insertions took place in the same clinic where prenatal appointments are conducted, a location already familiar to most women.

According to the Ministry of Health, only 5% of women experience moderate or severe levels of pain^(15)^, which does not align with the findings of this and other studies that focused on this aspect from the women’s perspective. IUD insertion appears to be a painful procedure, and strategies to minimize pain should be addressed in manuals and guidelines, aiming to raise awareness among professionals. Thus, there is still a knowledge gap regarding pain management during the IUD insertion procedure, as it is perceived as painful by women, and currently, there is no scientific evidence supporting the benefits of pharmacological interventions^(35)^.

Copper IUD users report high satisfaction with the method, as observed in Zambia, where, for example, 94.1% of women reported being satisfied or very satisfied with the method^(36)^; in the United States, where 80% of users were satisfied^(32)^; in São Paulo, where 94.7% of them were highly satisfied^(37)^; and in Recife, where 77.8% of nulliparous and 76.7% of multiparous women considered themselves completely satisfied^(38)^. These data justify the provision and access to the IUD when there is an intention to use it.

Regarding method adaptation, it was observed that changes in menstrual flow and cramps were the main complaints among users, as expected, since these are precisely the most common side effects^(39)^.

Regarding the sociodemographic characteristics and obstetric history of the IUD users who had their insertion in a PHBC setting, a higher prevalence was observed among young women, aged between 25 and 29 years, with at least one prior pregnancy, self-identified as Black, employed, and possessing a high level of education. These findings align with a study conducted in the cities of São Paulo, Aracaju, and Cuiabá, where young women with children were more inclined to express interest in using the IUD compared to those without children^(40)^. Moreover, it was found that women with higher education were more knowledgeable about the method and, consequently, more likely to seek it out^(38,40)^.

The percentage of women who returned to the service for follow-up consultations was higher than the findings in the Paraná study (57%)^(29)^ and in Australia (53%)^(25)^, but similar to the findings in India (63%). It appears that the demand for PHBC services arises from women’s own desires during pregnancy and childbirth, which can be a vulnerable period. Providing support during this time can have an impact on their connection with the service, resulting in increased participation in appointments^(41)^.

One strategy to enhance the rate of return for these women to the service would be to actively reach out to them through telephone contact and offer a channel for them to freely seek the service in case of any complications. Additionally, it is crucial to train the teams to deliver empathetic care that nurtures a strong rapport. Another strategy would be to leverage digital technologies for disseminating health-related content and conducting video calls for follow-up consultations.

### Study limitations

Among the limitations of the study, one notable aspect is the analysis of pain, as this information was not obtained immediately after the insertion procedure. Therefore, memory bias and adaptation to the method may have influenced women’s perception. However, pain is rarely assessed in studies on copper IUDs, and our findings demonstrate the importance of investing in measures to alleviate pain during insertion, as well as conducting studies that evaluate strategies for its control and prevention. Approximately one-third of the women did not return to the institution for follow-up consultation, which prevented data collection regarding potential complications and outcomes in this group. Another relevant point is the sample size of the study and the fact that it is a non-probabilistic sample. Therefore, it is not possible to generalize the findings, although the results demonstrate favorable outcomes of IUD insertions performed by nurses and midwives.

### Contributions to the field of nursing, health, or public policy

The results of this study provide insights for the development and implementation of public policies regarding sexual and reproductive health. Additionally, they indicate the inclusion of midwives and nurses in reproductive planning and contraception care, including copper IUD insertion and management. This is because the outcomes of copper IUD insertion by these professionals did not differ from those when other professionals, including physicians, were responsible for the insertion.

## CONCLUSIONS

The study has demonstrated the safety of copper IUD insertion by trained nurses and midwives. Furthermore, the outcomes are comparable to those observed in various other studies conducted among women whose IUDs were inserted by physicians. Nurses and midwives who have received proper training are capable of performing the procedure safely and effectively, with rates of perforation, expulsion after 30 to 45 days of use, removal, and levels of pain similar to, or even lower than, those observed in other studies, both nationally and internationally. Moreover, these healthcare professionals have a strong focus on care and health promotion, which, in addition to expanding access to contraceptive methods, provides an opportunity to address other issues related to women’s health and sexual and reproductive rights.
